# Enhancer of zeste homolog 2 promotes hepatocellular cancer progression and chemoresistance by enhancing protein kinase B activation through microRNA-381-mediated SET domain bifurcated 1

**DOI:** 10.1080/21655979.2021.2023792

**Published:** 2022-02-19

**Authors:** Jingyang Zhou, Jinhui Che, Lu Xu, Weizhong Yang, Yunmei Li, Wuyuan Zhou, Shubing Zou

**Affiliations:** a Queen Mary School, Medical Department, Nanchang University, Nanchang, P.R. China; b Department of Hepatobillary Surgery, The Affiliated Suzhou Science and Technology Town Hospital of Nanjing Medical University, Suzhou, P.R. China; c Department of Hepatopancreatobillary Surgery, Xuzhou Cancer Hospital, Xuzhou, P.R. China; d Department of Hepatopancreatobillary Surgery, The Second Affiliated Hospital of Nanchang University, Nanchang, P.R. China

**Keywords:** Hepatocellular cancer, enhancer of zeste homolog 2, microRNA-381, SET domain bifurcated 1, AKT pathway, chemoresistance

## Abstract

Metastasis and chemoresistance are the leading causes of death in patients with hepatocellular carcinoma (HCC). microRNAs (miRNAs or miRs) may be useful as diagnostic, therapeutic and prognostic markers for HCC. In this study, we set out to investigate the possible role of miR-381 in HCC development and chemoresistance along with the related mechanism. Microarray-based gene expression profiling was carried out to analyze the expression of SET domain bifurcated 1 (SETDB1) and histone methyltransferase enhancer of zeste homolog 2 (EZH2) followed by validation in clinical HCC tissues and cells. The potential binding between miR-381 and SETDB1 was found and verified. Then, the role of SETDB1 in HCC in relation to miR-381 and protein kinase B (AKT) pathway was explored through gain- and loss-of-function approaches. After expression determination of EZH2, SETDB1, miR-381, and AKT pathway-related factors, their reactions were analyzed and their functional roles in HCC progression and chemoresistance were investigated *in vitro* and *in vivo*. SETDB1 was aberrantly upregulated in clinical HCC tissues and cells. This upregulation activated AKT pathway by promoting its tri-methylation on K64. SETDB1 promoted the proliferation, migration and chemoresistance through the AKT pathway in HCC cells. In a xenograft mouse model, SETDB1 promoted HCC cell tumorigenesis *in vivo* by activating the AKT pathway. Furthermore, EZH2 suppressed miR-381 by catalyzing the activity of H3K27me3 on its promoter region. In conclusion, EZH2 suppressed miR-381 expression by promoting H3K27me3 activity on its promoter region to facilitate SETDB1 expression, thereby activating the AKT pathway to promote hepatocarcinogenesis and chemoresistance.

## 1. Introduction

Hepatocellular cancer (HCC) is one of the most common kinds of cancer, which brings high mortality in the worldwide [1]. Accumulating evidence reveals that chronic liver disease, such as chronic viral hepatitis types B and C, abuse of alcohol, aflatoxin exposure, and nonalcoholic fatty liver disease, are the major factors that trigger HCC [2–4]. More importantly, distant metastasis and chemoresistance are the leading cause of death among HCC patients [5]. Thus, obtaining a better understanding of molecular mechanism of HCC metastasis and chemoresistance is critical for improved HCC therapy.

Aberrantly activated phosphoinositol 3 kinase-protein kinase B (PI3K-AKT) is a frequent finding in various cancers including HCC [6,7]. Moreover, constituently activated AKT pathway is tightly associated with HCC metastasis and chemoresistance [8,9]. SET domain bifurcated 1 (SETDB1) has been reported to activate AKT by promoting its methylation [10] and high expression of SETDB1 indicates high metastatic capacity and chemoresistance [11,12]. Indeed, the majority of the human genome does not encode proteins, but serves other functions including the expression of non-coding RNAs such as microRNAs (miRNAs or miRs), long noncoding RNAs, or circular RNAs [13]. miRNAs have been reported to be involved in diverse biological process, including cell cycle transition, apoptosis, proliferation and chemoresistance [14–16]. For instance, miR-381 has been reported to repress SETDB1 to inhibit breast cancer proliferation [17]. However, its role in HCC progression still remains largely unknown. Interestingly, enhancer of zeste homolog 2 (EZH2), an epigenetic regulator, catalyzes histone H3 Lys27 trimethylation (H3K27me3) activity to suppress gene expression [18]. A previous report revealed that EZH2 contributed to breast cancer chemoresistance by suppressing miR-381 expression via catalyzing H3K27me3 on its promoter region [19]. According to these lines of evidence, we hypothesize that EZH2 might be involved in the progression and chemoresistance of HCC with interaction with miR-381, SETDB1 and AKT. Thus, we enrolled HCC clinical samples and combined with *in vitro* and *in vivo* experiments to determine if the aforementioned hypothesis was valid so as to achieve a deeper understanding of the mechanism underlying the progression and chemoresistance of HCC.

## 2. Materials and methods

### 2.1. Ethical approval

The experiment was approved by the Ethics Committee of Xuzhou Cancer Hospital (approval number: 2017–02-018-K01) and in accordance with stipulations of the *Declaration of Helsinki*, and written informed consent was signed by all included individuals. Experimental procedures involving animals were approved by the Animal Ethics Committee of Xuzhou Cancer Hospital.

### 2.2. Microarray-based gene expression profiling

HCC-related mRNA expression dataset GSE89377 was obtained from the Gene Expression Omnibus (GEO) database, including 40 HCC samples and 13 normal samples. Differential analysis was performed using the R “limma” package and the differentially expressed genes were selected by the criteria of |logFC| > 0.8 and *p*-value < 0.01. Analysis of EZH2 expression in HCC samples was performed using the GEPIA (http://gepia.cancer-pku.cn/index.html) database. Downstream target genes of miR-381 were then predicted via the starBase database (http://starbase.sysu.edu.cn/index.php) and miRWalk database (http://mirwalk.umm.uni-heidelberg.de/). Finally, the intersection was made between database prediction results and the significantly up-regulated mRNA obtained from the GSE89377 dataset.

### 2.3. HCC clinical samples

A total of 52 patients with HCC (aged 34–72 years old) confirmed by needle aspiration biopsy and pathological diagnosis at the Department of Hepatopancreatobillary Surgery of Xuzhou Cancer Hospital from September 2018 to February 2020 was selected for HCC sample collection. The characteristics of HCC patients are shown in Table S1. Besides, 45 patients with hepatic benign hyperplasia (aged 28–67 years old) were selected during the same period for collection of normal liver tissues through biopsy. Inclusion criteria were as follows: patients were diagnosed with HCC according to World Health Organization criteria; all tumor specimens were confirmed to contain more than 80% tumor cells in pathological sections by pathological examination; no anticancer treatment was given before surgery; complete resection of all tumor nodules was confirmed by pathological examination of surface free tumor tissues; patients had complete clinicopathological and follow-up data. Patients died from non-HCC or accident were excluded. According to the classification of serum alpha-fetoprotein (AFP) levels, among 52 HCC patients, 5 patients had AFP levels < 25.0 μg/mL, 26 had AFP levels between 25.0 and 200.0 μg/mL, and 21 had AFP levels > 200.0 μg/mL; among 45 patients with hepatic benign hyperplasia, 25 had AFP levels < 25.0 μg/mL, 13 had AFP levels between 25.0 and 200.0 μg/mL, and 7 had AFP levels > 200.0 μg/mL.

### 2.4. Cell culture

HCC cell lines Huh7, Hep3B, MHCC-97 H, and HB611 were purchased from Shanghai Institutes for Biological Sciences, Chinese Academy of Sciences (Shanghai, China). Human normal liver cell line MIHA was purchased from Fenghui Biotech Co., Ltd. (Human, China; CL0469; https://www.fenghbio.cn/). CDDP resistant Huh7 (Huh7/CDDP) cells were obtained from Shanghai Yaji Biotechnology Co., Ltd (Shanghai, China). All the cell lines were cultured in Dulbecco’s modified Eagle’s medium (DMEM) (Gibco, Carlsbad, CA, USA) appended to 10% fetal bovine serum (FBS; Thermo Fisher Science, Inc., Waltham, MA, USA), 100 U/mL penicillin and 100 µg/mL streptomycin, and maintained at 37°C in a saturated humidity atmosphere containing 95% air and 5% CO_2_.

### 2.5. Cell transfection and grouping

pLV-EGFP-N (Lentiviral overexpression vector) and pSIH1-H1-copGFP (lentiviral fluorescence silence vector) were obtained from Shanghai GenePharma Co., (Shanghai, China) and applied for SETDB1 overexpression, EZH2 knockdown, or rescue expression with silenced EZH2 and SETDB1. Overexpression-negative control (oe-NC), short hairpin RNA (sh)-NC, sh-/oe-EZH2, and sh-/oe-SETDB1 vectors obtained from GenePharma were used for cell transfection.

293 T cells were transfected with expression vectors using Lipofectamine 3000 transfection reagent (Invitrogen Inc., Carlsbad, CA, USA). After 48 h, the supernatant was collected and centrifuged to remove cell debris, with lentivirus obtained. During transduction, Hep3B and Huh7 cells were seeded in a 6-well plate, 2 mL per well. When reaching 50% confluence, Hep3B and Huh7 cells were transduced with lentivirus carrying overexpression vectors and incubated with the prepared lentiviral supernatant (concentration higher than 10^7^ TU/mL). After 48 h of incubation, G418 was used to screen the stably transduced cells for more than 2 weeks. The protein expression was identified by reverse transcription quantitative polymerase chain reaction (RT-qPCR) or enzyme-linked immunosorbent assay (ELISA).

### 2.6. 3-(4,5-dimethylthiazol-2-yl)-2, 5-diphenyltetrazolium bromide (MTT) assay

Huh7 or Hep3B cells were seeded into 96-well plates at a density of 1 × 10^4^ cells/well, cultured for 24 h, and then treated with gradient dose of CDDP (0.2, 0.5, 1.0, 2.0, 5.0, and 10.0 μg/mL) for 48 h. Subsequently, 10 μL of MTT (5 mg/mL) was added to each well. After incubation for 3 h, optical density value of each well was determined at 490 nm on a plate reader. The cell survival rate was calculated. The cell survival rate of the drug concentration of 0 μg/mL (control group) was 100%, and the cell survival rate of the remaining groups was analyzed. In three independent experiments, the half maximal inhibitory concentration (IC_50_) value was calculated as the half-lethal dose [20].

### 2.7. Transwell assay

Transwell chamber (8 μm pore size; Corning, USA) was used for *in vitro* cell migration [21]. The migrating cells were counted under an inverted microscope (Olympus Corporation, Tokyo, Japan).

### 2.8. RT-qPCR

Total RNA was extracted from tissues and cells using TRIzol reagent (15596026, Invitrogen, CA, USA). RNA was then reversely transcribed into complementary DNA (cDNA) according to the instructions of a Reverse Transcription kit (RR047A, Takara, Japan). For miRNA detection, PolyA Tailing Reverse Transcription Kit (B532451, Sangon Biotech Co., Ltd., Shanghai, China; containing universal PCR primer R and U6 universal PCR primer R) was used for reverse transcription, with PolyA-containing cDNA obtained. The concentration and purity of the RNA and cDNA quality were determined using the S23A visible spectrophotometer at the wavelength of 340–950 nm. RT-qPCR was performed using SYBR Premix Ex TaqTM II (Perfect Real Time) kit (DRR081, Takara, Japan) on an ABI 7500 quantitative PCR instrument. The amount of reagents used in real-time PCR was as follows: SYBR premix Ex Taq: 10 μL/sample, PCR forward primer (20 μM): 0.2 μL/sample, PCR reverse primer (20 μM): 0.2 μL/sample, Rox Reference dye II: 0.2 μL/sample, cDNA: 2 μL/sample, dH_2_O: 7.2 μL/sample. The reaction conditions of RT-qPCR were consisted of pre-denaturation at 95°C for 2 min, denaturation at 95°C for 15 s and annealing at 60°C for 45 s, with a total of 45 cycles. Glyceraldehyde-3-phosphate dehydrogenase (GAPDH) served as the internal reference for EZH2, SETDB1 and AKT, and U6 served as reference for miR-381. The 2^−ΔΔCt^ method was employed to calculate the ratio of the relative expression of a target gene in the experimental group to that in the control group. Three independent experiments were conducted. The primers used are listed in Table S2.

### 2.9. ELISA

Cells were collected from each group, washed 2–3 times with pre-cooled phosphate-buffered saline (PBS) buffer, and lysed with protein extraction lysis buffer to extract total protein. ELISA was used to detect the expression of SETDB1, EZH2, AKT, mammalian target of rapamycin (mTOR), proliferation and migration marker proteins, cell cycle and apoptosis marker proteins, levels of AKT phosphorylation and methylation, and mTOR phosphorylation level. Next, gradient standard and test sample (each for 100 μL) was added to each well and left to stand at 37°C (a wet box or sealing) for 30 min. Following washing 4 times with washing solution, cells were added with 100 μL of enzyme conjugate at 37°C (a wet box or sealing) for 30 min. After washing 4 times again, one drop of developer A and B, respectively, was supplemented to the cells, mixed, and reacted at 37°C (a wet box or sealing) for 15 min in the dark, and then the reaction was halted by one drop of stop solution. Finally, the result was observed with naked eyes and the optical density was measured at 450 nm.

### 2.10. Immunoprecipitation (IP)

Cells were lysed by incubating with IP lysis buffer (Beyotime) containing protease inhibitor for 30 min on ice and then subjected to centrifugation for 30 s at 2000 g. A small portion of the lysates was subjected to Western blot analysis and the remainder was incubated with anti-AKT antibody (ab179463, 1:100, Abcam) and protein A/G beads overnight at 4°C. After incubation, the beads were washed 3–4 times, mixed with loading buffer and then subjected to ELISA [22].

### 2.11. In vitro methylation assay

HEK293T cells were transfected with Flag-SETDB1 plasmids or corresponding Flag-NC plasmids and incubated with S-adenosylmethionine (Abcam) and HA-AKT or corresponding HA-NC plasmid at 30°C for 1–1.5 h. The reaction was then terminated by adding an appropriate amount of protein loading buffer, and then the cells were heated at 100°C for 5 min and subjected to ELISA [22].

### 2.12. Chromatin immunoprecipitation (ChIP)

ChIP assay was performed using an EZ-Magna ChIP Kit (EMD Millipore). Huh7 cells were crosslinked by 1% formaldehyde for 10 min and incubated with 0.125 M glycine for 5 min. The cells were then rinsed twice with PBS, lysed with ChIP lysis buffer (150 mM NaCl, 50 mM Tris [pH 7.5], 5 mM EDTA, 0.005% NP40, and 0.01% Triton X-100) and sonicated to generate 200–1000 bp chromatin fragments. For each group, 100 μL of supernatant (DNA fragment) was added to 900 μL of ChIP Dilution Buffer, 20 μL of 50× PIC and 60 μL of ProteinA Agarose/Salmon Sperm DNA. After that, the mixture was left to stand at 4°C for 10 min and then centrifuged at 200 g for 1 min. The supernatant was taken and 20 μL was used as input which was incubated with rabbit anti-EZH2 antibody overnight in the experimental group and 1 μL of rabbit anti-IgG in the negative control group. A 60 μL volume of protein A agarose/salmon sperm DNA was added to each tube for 2 h at 4°C. After that, the sample was washed and precipitated with 1 mL of low-salt buffer, high-salt buffer, and LiCl solution, TE (twice), respectively. Each tube was eluted twice with 250 mL ChIP Wash Buffer and decrosslinked by addition of 20 mL 5 M NaCl. Thereafter, DNA was recovered and the miR-381 promoter was quantified in the complex by RT-qPCR [23]. The miR-381 promoter primer sequence is shown in Table S2.

### 2.13. Dual luciferase assay

Synthesized wild type (WT) or binding sites mutant 3’untranslated region (3’UTR) of SETDB1 fragments (WT-SETDB1 and mutant type [Mut]-SETDB1) was ligated to the psiCHECK2 vector. Sequenced WT- or Mut-SETDB1 construct was co-transfected with miR-381- or NC-mimic into 293 T cells. Cell lysates were collected after transfected for 48 h and used for luciferase activity detection in a Glomax20/20 luminometer (Promega) by Luciferase Detection Kit (K801, Biovision). This experiment was repeated at least 3 times independently. The targeting regulation between EZH2 and miR-381 was analyzed in the same steps as above, but with the construction of WT-miR-381 and Mut-miR-381 promoter in the dual luciferase reporter vector [23].

### 2.14. Flow cytometry

After transfection for 48 h, the Huh7/CDDP cells were collected and digested with 0.25% trypsin, with the cell concentration adjusted to 1 × 10^6^ cells/mL. Cells (1 × 10^6^) were then centrifuged at 800 g for 10 min with the supernatant discarded. Next, the cells were fixed with pre-chilled 70% ethanol at 4°C overnight, and washed twice with PBS. Then, 100 μL of cell suspension was added with 0.5 mL containing 50 µg/mL RNAase propidium iodide (PI) solution (40710ES03, Shanghai Qianduan Biotechnology Co., Ltd., Shanghai, China), and filtered through a 100 mesh nylon net after incubation in a dark room for 30 min. A flow cytometer (BD, FL, NJ, USA; 488 nm and 635 nm dual-space three-dimensional excitation, four-color fluorescence detection system) was used to record the red fluorescence at an excitation wavelength of 488 nm for detection of cell cycles and count the number of cells in different cycles [24].

### 2.15. HCC xenograft tumor mouse model

BALB/c nude mice (n = 60), obtained from Servicebio (Wuhan, China), were given a standard diet and maintained in a specific pathogen-free animal facility. Mice were divided into sh-NC + oe-NC, sh-SETDB1 + oe-NC, sh-NC + oe-AKT, sh-SETDB1 + oe-AKT, sh-EZH2 + oe-NC, and sh-EZH2 + oe-AKT, mimic-miR-NC, and mimic-miR-381 (ten mice per group) groups. Stably transfected cells were obtained, incubated *in vitro* for 3–7 days and then made into 50 μL of 1 × 10^6^ single-cell suspension, which was combined with 50 μL of Matrigel solution and injected in the right upper dorsal region of nude mice (10 per group). The size of the tumor was measured with a vernier caliper every week starting from day 7 of transplantation. Data were recorded and tumor volume was calculated as: tumor volume (V) = 0.8 × 2/3 × D1^2^ × D2 (D1, D2 are the longest and shortest diameters perpendicular to each other, respectively). After that, statistical changes in the weight of the tumor body were recorded. In an experiment to detect CDDP resistance in a xenograft model, CDDP was formulated into a solution of 2.5 mg/mL in saline and administered at a dose of 25 mg/kg in constructed nude mice intraperitoneally (0.1 mL/kg) once every other day for five consecutive doses, and data were recorded as above. Mice were executed by euthanasia with an intravenous three-fold overdose of 3% sodium pentobarbital (P3761, Sigma, USA).

### 2.16. Immunohistochemistry

Tumors were removed from the euthanized nude mice, dried, fixed in 4% paraformaldehyde solution, routinely paraffin-embedded, sectioned into 4 μm serial sections, and subjected to antigen retrieval. The sections were rinsed with PBS and blocked by normal goat serum. HistostainTMSP-9000 immunohistochemical staining kit (Zymed) was used to stain the sections. The sections were incubated with indicated primary antibodies at 4°C overnight. After rinsing with PBS, sections were incubated with secondary antibody goat anti-rabbit (ab6721, 1:1000, Abcam) and goat anti-mouse (ab6728, 1:1000, Abcam). The results were visualized after addition of diaminobenzidine for 5 to 10 min, with the staining time adjusted by monitoring under the microscope. After re-staining with hematoxylin for 1 min, the sections were mounted and photographed. Five representative high-frequency fields of view (NIKON, Japan) were selected for observation and counting, with the cytoplasm being positive if brown or yellow colored. The antibodies used were: SETDB1 mouse antibody (ab107225, 1:100, Abcam), EZH2 rabbit antibody (ab186006, 1:100, Abcam), AKT rabbit antibody (ab179463, 1:250, Abcam), PT308-AKT rabbit antibody (ab38449, 1:100, Abcam), PS473-AKT rabbit antibody (ab81283, 1:100, Abcam), and AKT K140me3 rabbit antibody (1:1000, CST).

### 2.17. Hematoxylin-eosin (HE) staining

The formaldehyde-fixed specimens were rinsed with distilled water for 1 h, immersed, washed with saline, fixed in 4% paraformaldehyde for 30–50 min, washed, dehydrated, cleared, soaked in wax, and sectioned. The tissue sections were then dried at 45°C, dewaxed, and washed with distilled water for 5 min. Then, the tissue sections were stained with hematoxylin for 5 min, washed under running tap water for 3 s, treated with 1% ethanol hydrochloride for 3 s, and stained with 5% eosin stain for about 3 min. Thereafter, the sections were dehydrated, cleared, sealed, observed and photographed using a normal light microscope.

### 2.18. Statistical analysis

Data are shown as the mean ± standard deviation from at least three independent experiments performed. Firstly, the normality and homogeneity of variance were checked. Comparisons were implemented utilizing unpaired t-test when only two groups (data with normal distribution and even variance) were compared, or by Tukey’s test-corrected one-way analysis of variance (ANOVA) when more than two groups were compared. Variables were analyzed at different time points using Bonferroni-corrected repeated measures ANOVA. All statistical analyses were implemented utilizing SPSS 21.0 software (IBM, Armonk, NY, USA), with two-tailed *p* < 0.05 as a level of statistical significance.

## 3. Results

In the current study, we focused on the possible role of miR-381 in HCC development and chemoresistance along with the related mechanism. Through a series of *in vitro* and *in vivo* experiments, we provided evidence that EZH2 suppressed miR-381 expression by promoting H3K27me3 activity on its promoter to facilitate SETDB1 expression, thereby activating the AKT pathway to promote hepatocarcinogenesis and chemoresistance.

### 3.1. SETDB1 is highly expressed in both HCC tissues and cells

**Figure 1.**
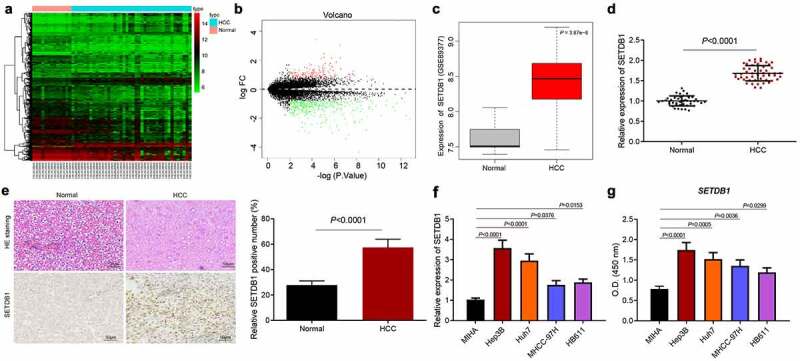
SETDB1 is aberrantly overexpressed in clinical HCC tissue samples and cell lines. a, a heatmap of differentially expressed genes in HCC samples screened from the GSE89377 microarray dataset; the abscissa indicates the sample number, the ordinate indicates the gene name, and each small box indicates the expression of one gene in a sample. b, a volcano plot of differentially expressed genes in HCC samples screened from the GSE89377 microarray dataset. Black portion indicates no significant difference, green indicates downregulated mRNAs, while red indicates upregulated genes. c, expression of SETDB1 expression in HCC samples and normal samples from the GSE89377 microarray dataset. d, SETDB1 expression in HCC tissues (N = 52) and normal liver tissues (N = 45) determined by RT-qPCR, * *p* < 0.05 compared to normal liver tissues. e, SETDB1 expression in HCC biopsy specimens determined by IHC and histopathological changes determined by HE staining (× 200), * *p* < 0.05 compared to normal liver tissues. f, SETDB1 expression in HCC cells determined by RT-qPCR, * *p* < 0.05 compared to MIHA cells. G, SETDB1 protein levels in HCC cells determined by ELISA and quantified by Image j, * *p* < 0.05 compared to MIHA cells. The cell experiment was repeated three times.

SETDB1 is a histone methyltransferase that has been reported to be involved in progression of diverse cancer types [10,22]. However, its role in HCC had not been well studied. We first analyzed the expression of SETDB1 in the HCC-related microarray dataset GSE89377, which revealed an aberrant upregulation of SETDB1 in HCC samples (Figure 1(a-c)). Meanwhile, RT-qPCR results showed that SETDB1 was highly expressed in HCC clinical tissue samples compared to normal liver tissue samples (Figure 1(d)). Consistently, IHC analysis results revealed elevated SETDB1 protein levels in HCC tissues in comparison with that in normal tissues (Figure 1(e)). HE staining results displayed that, in normal liver tissues, the cells were arranged orderly and the morphology was normal, while in HCC tissues, the cells were arranged in a disorderly and irregular manner (Figure 1(e)). Furthermore, the results of RT-qPCR and ELISA suggested that SETDB1 expression was significantly upregulated in HCC cells in comparison with normal liver cells MIHA, among which Hep3B and Huh7 cells exhibited the highest SETDB1 expression, and were chose for further studies (figure 1(f,g)). The above results indicated that SETDB1 was abundantly expressed in HCC tissues and cells.

### 3.2. SETDB1 activates AKT pathway by promoting AKT methylation

**Figure 2.**
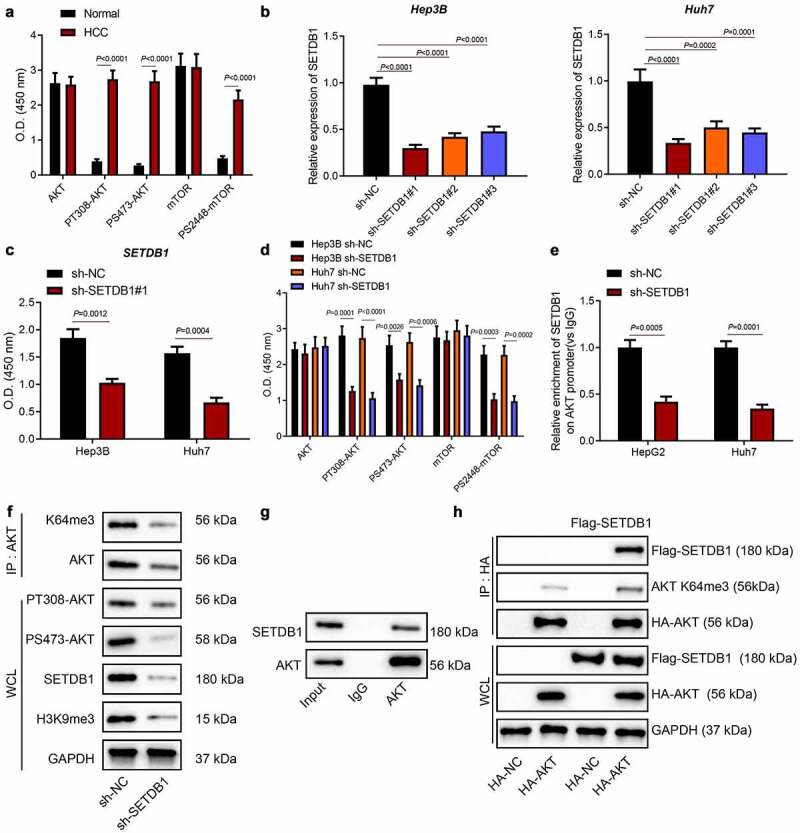
SETDB1 activates the AKT pathway by promoting AKT methylation. a, expression of the AKT pathway-related factors AKT and mTOR and their phosphorylation levels in HCC tissues determined by ELISA, * *p* < 0.05 compared to normal liver tissues. b, knockdown efficiency of sh-SETDB1 in Hep3B and Huh7 cells determined by RT-qPCR, * *p* < 0.05 compared to sh-NC. c, knockdown efficiency of sh-SETDB1 in Hep3B and Huh7 cells determined by ELISA, * *p* < 0.05 compared to sh-NC. D, AKT and mTOR phosphorylation levels in Hep3B and Huh7 cells with sh-SETDB1 determined by ELISA, * *p* < 0.05 compared to sh-NC. e, ChIP test for binding of SETDB1 to the AKT promoter region. f, the effect of SETDB1 expression on AKT methylation determined by IP and Western blot. g, interaction between SETDB1 and AKT determined by IP. h, AKT K64me3 determined by IP. The cell experiment was repeated three times.

Previous reports revealed that SETDB1 activated AKT pathway to promote malignant melanoma progression by methylating AKT [10,22]. On the other hand, PI3K/AKT as a classic pathway is also involved in HCC progression [25]. To further investigate whether SETDB1 promoted HCC progression by activating AKT, we employed ELISA to determine the expression of the AKT pathway-related factors AKT and mTOR and their phosphorylation levels. The results revealed that, although the total levels of these two kinases were not significantly different between HCC and normal tissues, AKT-T308, AKT-S473 and mTOR-S2448 levels were aberrantly elevated in HCC tissues (Figure 2(a)). Next, we knocked down SETDB1 using three independent shRNA sequences and validated the knockdown efficiency by RT-qPCR and ELISA, which showed that the sh-SETDB1#1 sequence achieved the best knockdown efficiency (Figure 2(b,c)). Based on this, sh-SETDB1#1 was chosen for following experiments.

Consistently, we found that SETDB1 silencing significantly suppressed AKT and mTOR activation without affecting their total levels in HCC cells (Figure 2(d)). Moreover, ChIP results indicated that depletion of SETDB1 significantly reduced the enrichment of SETDB1 in the AKT promoter (Figure 2(e)). Also, depletion of SETDB1 significantly reduced H3K9me3 expression (Figure 2(f)). The IP experiment for detecting the targeting effect of SETDB1 and AKT demonstrated that, compared with IgG, SETDB1 was distinctly present in the pull-down product using AKT-specific antibodies (Figure 2(g)). Furthermore, after overexpression of Flag-SETDB1, the expression of AKT K64me3 in the pull-downs with AKT-specific antibodies was significantly up-regulated compared with overexpression of Flag-NC, proving that overexpression of SETDB1 promoted the trimethylation of AKT on lysine 64 (Figure 2(h)). Taken together, our study revealed that SETDB1 facilitated AKT activation by promoting phosphorylation of AKT and mTOR.

### 3.3. SETDB1 promotes cell proliferation, migration, and chemoresistance by activating AKT pathway

**Figure 3.**
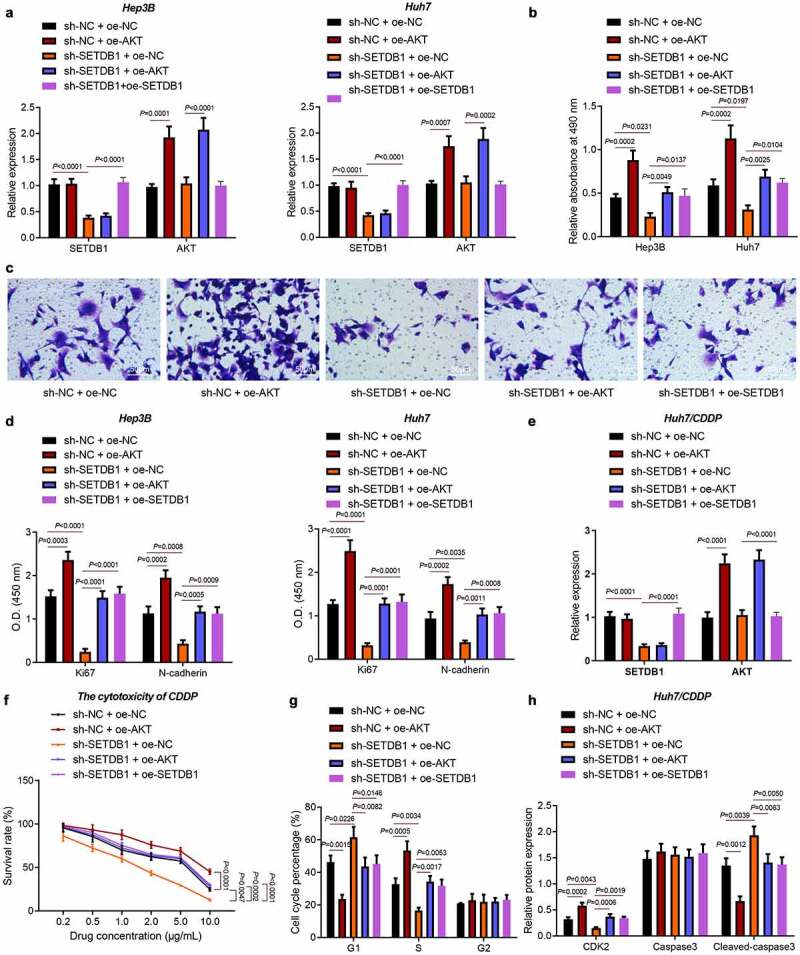
SETDB1 promotes HCC cell proliferation, migration and chemoresistance by activating AKT pathway. Hep3B and Huh7 cells were treated with sh-SETDB1 + oe-NC, sh-NC + oe-AKT, sh-SETDB1 + oe-AKT or sh-SETDB1 + oe-SETDB1. a, SETDB1 and AKT expression in Hep3B and Huh7 cells determined by RT-qPCR. b, proliferation of Hep3B and Huh7 cells determined by MTT; c, migration of Hep3B and Huh7 cells determined by Transwell assay (× 200). d, expression of proliferation- and cell cycle-related factors in Hep3B and Huh7 cells determined by ELISA. Huh7/CDDP cells were treated with sh-SETDB1 + oe-NC, sh-NC + oe-AKT, sh-SETDB1 + oe-AKT or sh-SETDB1 + oe-SETDB1. e, SETDB1 and AKT expression in Huh7/CDDP cells determined by RT-qPCR. f, sensitivity to CDDP treatment determined by MTT assay. g, cell cycle distribution determined by flow cytometry. h, expression of cell cycle and apoptosis related factors determined by ELISA. * *p* < 0.05, compared to sh-NC+ oe-NC, # *p* < 0.05, compare to sh-SETDB1 + oe-NC. The cell experiment was repeated three times.

Next, we aimed to investigate whether SETDB1 promoted HCC progression by activating the AKT pathway. The results of RT-qPCR revealed an increase in the expression of AKT in Hep3B and Huh7 cells treated with sh-NC + oe-AKT and a decline was noted in the expression of SETDB1 in cells treated with sh-SETDB1 + oe-NC. In addition, the expression of AKT was higher in cells treated with sh-SETDB1 + oe-AKT than that in cells treated with sh-SETDB1 + oe-NC. The down-regulated SETDB1 expression could be rescued by further oe-SETDB1 treatment (Figure 3(a)). Moreover, the results of MTT and Transwell assays showed that SETDB1 silencing significantly suppressed HCC cell proliferation and migration, which was reversed by oe-AKT or oe-SETDB1 (Figure 3(b,c)). Meanwhile, SETDB1 knockdown reduced expression of the proliferation and migration markers KI67 and N-cadherin, while their expression was rescued by treatment with oe-AKT or oe-SETDB1 (Figure 3(d)).

Next, we turned to explore the role of SETDB1 in CDDP resistance. We overexpressed AKT in the SETDB1 depleted CDDP-resistant cell-line Huh7/CDDP (Figure 3(e)). After cells were treated with gradient doses of CDDP, we employed the MTT assay to validate the effect of SETDB1 depletion on CDDP sensitivity. Results indicated that IC_50_ in control cells was 4.909 µg/mL, IC_50_ in sh-SETDB1-treated cells was 9.625 µg/mL, IC_50_ in AKT overexpressing cells was 1.702 µg/mL, IC_50_ in oe-AKT + sh-SETDB1-treated cells was 5.017 µg/mL, and IC_50_ in sh-SETDB1 + oe-SETDB1-treated cells was 5.141 µg/mL. These values indicated that SETDB1 silencing significantly increased CDDP sensitivity in CDDP-resistant cells, which was rescued by oe-AKT or oe-SETDB1 (Figure 3(f)).

Besides, CDDP treatment in SETDB1 depleted cells led to more cells arrested at the G1 phase, fewer cells arrested at the S phase and no changes in cells arrested at the G2 phase. In addition, AKT or SETDB1 overexpression caused reduced cells arrested at the G1 phase, increased cells arrested at the S phase, and no changes in cells arrested at the G2 phase (Figure 3(g)). Consistently, ELISA results presented that SETDB1 depletion significantly reduced the expression of CDK2, a marker for cell cycle while elevating that of cleaved caspase-3, a marker for cell apoptosis. These effects were reversed by ectopic AKT or SETDB1 expression (Figure 3(h)). Collectively, SETDB1 promoted HCC cell proliferation, migration and CDDP resistance by activating AKT pathway.

### 3.4. SETDB1 promotes tumorigenesis in HCC cell and chemoresistance by activating AKT pathway

**Figure 4.**
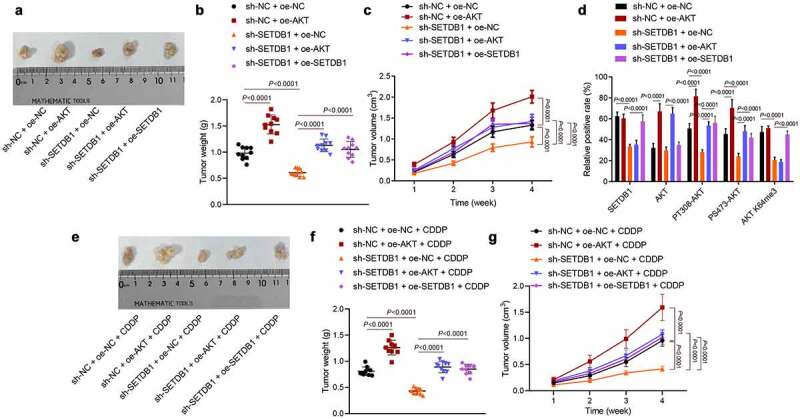
SETDB1 promotes HCC cells tumorigenesis and chemoresistance by activating AKT pathway. a, representative photos of xenograft tumors; b, weight of xenograft tumors, * *p* < 0.05, compared to sh-NC + oe-NC; # *p* < 0.05, compare to sh-SETDB1 + oe-NC. c, Xenograft tumor volume, * *p* < 0.05, compared to sh-NC + oe-NC; # *p* < 0.05, compare to sh-SETDB1 + oe-NC. d, SETDB1, AKT, K140me3 and its downstream phosphorylation status in xenograft tumors of each group determined by IHC, * *p* < 0.05, compared to sh-NC + oe-NC; # *p* < 0.05, compare to sh-SETDB1 + oe-NC. e, photos of each xenograft tumors under CDDP treatment. f, tumor weight under CDDP treatment, * *p* < 0.05, compared to sh-NC + oe-NC + CDDP; # *p* < 0.05, compare to sh-SETDB1 + oe-NC + CDDP. g, tumor volume under CDDP treatment, * *p* < 0.05, compared to sh-NC + oe-NC + CDDP; # *p* < 0.05, compare to sh-SETDB1 + oe-NC + CDDP. n = 10 for mice in each group.

Subsequently, we sought to determine whether SETDB1 regulated tumorigenesis and CDDP resistance *in vivo*. SETDB1 silencing significantly suppressed tumor volume and weight, while this effect was reversed by ectopic AKT expression; the effect of sh-SETDB1 treatment on tumor volume and weight could be reversed by overexpressing SETDB1 (Figure 4(a-c)). IHC analysis results showed that sh-SETDB1 significantly reduced SETDB1, AKT K64me3, PT308-AKT and PS473-AKT levels without affecting total AKT levels, while oe-AKT restored AKT, PT308-AKT and PS473-AKT levels, but showed no effect on the SETDB1 and AKT K64me3 levels. In addition, oe-SETDB1 elevated the SETDB1, AKT K64me3, PT308-AKT and PS473-AKT levels without affecting total AKT levels (Figure 4(d)). Furthermore, we utilized CDDP to treat the xenograft mice constructed as above, and observed tumor morphology, volume, and weight (Figure 4(e-g)). The results revealed that tumor volume and weight were significantly reduced under treatment with sh-SETDB1 + oe-NC + CDDP, the effect of which was abolished by AKT overexpression; treatment of oe-SETDB1 + oe-AKT + CDDP or sh-SETDB1 + oe-SETDB1 + CDDP could rescue the effect of sh-SETDB1 + oe-NC + CDDP on the tumor volume and weight. In summary, SETDB1 could activate AKT T308 and S308 phosphorylation sites by promoting methylation of AKT on K64me3 site, thereby accelerating the tumorigenesis and chemotherapy resistance of HCC cells in nude mice.

### 3.5. miR-381 suppresses AKT pathway by directly targeting SETDB1

**Figure 5.**
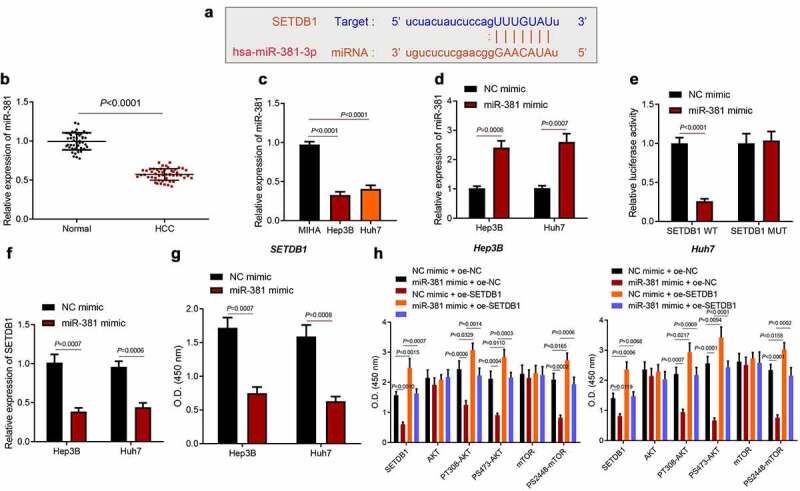
miR-381 targets SETDB1 to suppress AKT pathway. a, the binding sites of miR-381 on SETDB1 predicted by starBase. b, miR-381 expression in clinical HCC tissues (n = 52) and normal liver tissues (n = 45) determined by RT-qPCR, * *p* < 0.05, compared to normal liver tissues. c, miR-381 expression in HCC cell lines determined by RT-qPCR, * *p* < 0.05, compared to MIHA cell line. d, miR-381 overexpression efficiency in Hep3B and Huh7 cells determined by RT-qPCR, * *p* < 0.05, compared to NC-mimic. e, target relationship of miR-381 on SETDB1 validated by dual luciferase assay. F, SETDB1 expression in Hep3B and Huh7 cells after ectopic miR-381 expression determined by RT-qPCR, * *p* < 0.05, compared to NC-mimic. g, SETDB1 expression in Hep3B and Huh7 cells after ectopic miR-381 expression determined by ELISA, * *p* < 0.05, compared to NC-mimic. h, SETDB1, AKT, mTOR, PT308-AKT, PS2448-mTOR and PS473-AKT in Hep3B and Huh7 cells treated with miR-381 mimic or combined with os-SETDB1 determined by ELISA, * *p* < 0.05, compared to NC mimic + oe-NC; # *p* < 0.05, compared to miR-381 mimic + oe-NC. The cell experiment was repeated three times.

The upstream mechanism of SETDB1 in HCC was the next focus. starBase prediction results showed that miR-381 had binding sites in the 3’UTR of SETDB1 mRNA (Figure 5(a)). At the same time, we found through a literature search that miR-381 expression was significantly down-regulated in HCC [26]. Moreover, we analyzed miR-381 expression in clinical HCC tissue samples and found an aberrant downregulation of miR-381 (Figure 5(b)). Consistently, miR-381 was also found to be downregulated in HCC cell lines (Figure 5(c)). Based on this, we overexpressed miR-381 in Huh7 and Hep3B cells (Figure 5(d)) and used the dual luciferase assay to demonstrate that miR-381 targeted and bound to SETDB1 (Figure 5(e)). Moreover, we found that miR-381 overexpression significantly suppressed SETDB1 expression in the Huh7 and Hep3B cells (figure 5(f,g)).

To investigate whether miR-381 could suppress AKT activation through targeting SETDB1, we restored SETDB1 expression in miR-381 overexpressed cells and found that miR-381 overexpression indeed significantly suppressed AKT and mTOR activation while downregulating SETDB1 expression. On the other hand, restoration of SETDB1 abolished the effect of miR-381 overexpression on SETDB1 expression along with the AKT and mTOR activation (Figure 5(h)). Collectively, miR-381 suppressed AKT pathway by directly targeting SETDB1.

### 3.6. EZH2 reduces miR-381 to promote SETDB1 expression

**Figure 6.**
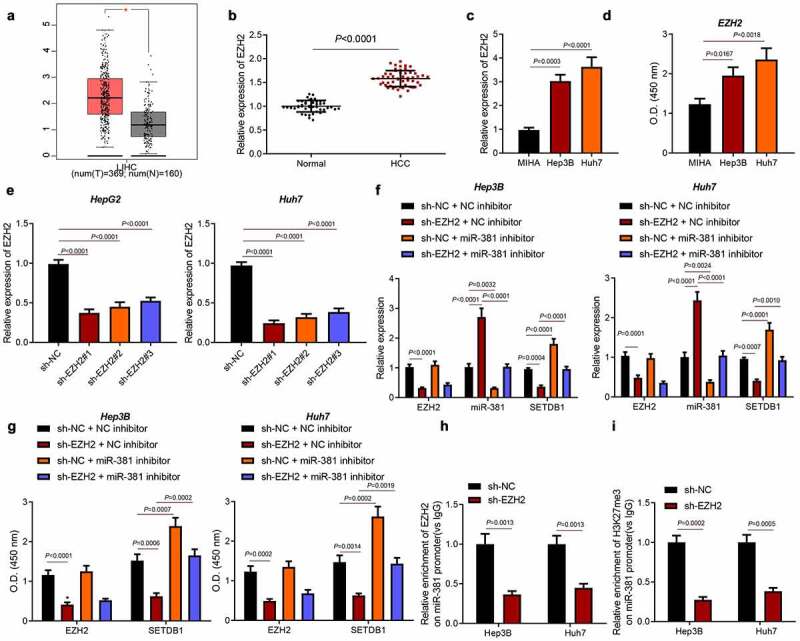
EZH2 represses miR-381 in an epigenetic way. a, EZH2 expression in HCC samples analyzed by GEPIA, * *p* < 0.05, compared to normal samples. b, EZH2 expression in HCC clinical samples (n = 52) and normal liver tissues (n = 45) determined by RT-qPCR, * *p* < 0.05, compared to normal liver tissues. c, EZH2 expression in HCC cell lines determined by RT-qPCR, * *p* < 0.05, compared to MIHA cells. d, EZH2 expression in HCC cell lines determined by ELISA, * *p* < 0.05, compared to MIHA cells. e, EZH2 knockdown efficiency determined by RT-qPCR, * *p* < 0.05, compared to sh-NC. f, miR-381 expression after EZH2 knockdown determined by RT-qPCR, * *p* < 0.05, compared to sh-NC + NC-inhibitor, # *p* < 0.05, compared to sh-EZH2 + NC-inhibitor. g, SETDB1 expression after EZH2 knockdown and miR-381 inhibitor treatment determined by ELISA, * *p* < 0.05, compared to sh-NC + NC-inhibitor; # *p* < 0.05, compared to sh-EZH2 + NC-inhibitor. H-I, EZH2 and H3K27me3 enrichment on the promoter region of miR-381 determined by ChIP-qPCR, * *p* < 0.05, compared to sh-NC. The cell experiment was repeated three times.

EZH2 had been widely studied in HCC [27] and has been reported to suppress miR-381 expression in an epigenetic manner [19]. To investigate whether EZH2 regulated HCC progression by suppressing miR-381, we firstly utilized an open access clinical database, GEPIA, to analyze EZH2 expression in HCC, which showed that EZH2 was highly expressed in HCC samples (Figure 6(a)). Consistently, an aberrant upregulation of EZH2 was also found in clinical HCC tissue samples and *in vitro* cell lines (Figure 6(b-d)).

Next, we silenced EZH2 in Hep3B and Huh7 cells by using three independent shRNAs and found that shEZH2#1 achieved the best knockdown efficiency (Figure 6(e)). Based on this, shEZH2#1 was chosen for further studies. We next detected SETDB1 and miR-381 expression in EZH2 silenced cells by RT-qPCR, which revealed that EZH2 knockdown led to significant upregulation of miR-381 and decreased SETDB1 expression. In contrast, simultaneous downregulation of EZH2 and miR-381 abrogated the effect of EZH2 silencing alone on the miR-381 and SETDB1 expression (Figure 6(f)). Moreover, the results of ELISA were consistent with those of RT-qPCR (Figure 6(g)). ChIP assay was then performed to validate the regulatory mechanism, which showed that EZH2 could directly bind to miR-381 promoter and that its silencing significantly reduced the enrichment of EZH2 on the miR-381 promoter region. Meanwhile, EZH2 silencing was found to reduce the enrichment of H3K27me3 on the miR-381 promoter region (Figure 6(h,i)). Collectively, EZH2 suppressed miR-381 expression to upregulate SETDB1 expression.

### 3.7. EZH2 promotes HCC cell migration, proliferation and chemoresistance by regulating the miR-381/SETDB1/AKT axis

**Figure 7.**
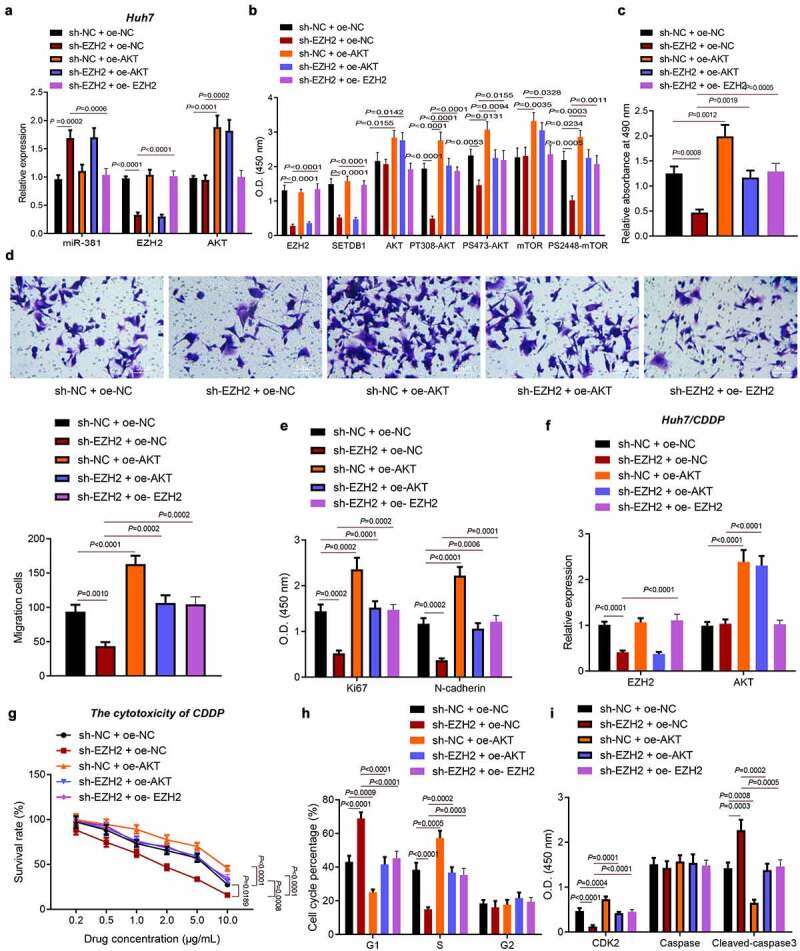
EZH2 promotes HCC cell proliferation, migration and chemoresistance by regulating the miR-381/SETDB1/AKT axis. a, EZH2 and AKT expression in Huh7 cells of each group were determined by RT-qPCR. b, EZH2, SETDB1, AKT, mTOR, PT308-AKT, PS2448-mTOR and PS473-AKT expression in Huh7 cells determined by ELISA. c, Huh7 cell proliferation rates determined by MTT. d, Huh7 cell migration capacity determined by Transwell assay (× 200). e, expression of proliferation and migration related factors in Huh7 cells determined by ELISA. F, EZH2 and AKT expression in Huh7/CDDP cells determined by RT-qPCR. g, CDDP sensitivity determined by MTT. h, Huh7/CDDP cell cycle distribution determined by flow cytometry. i, expression of cell cycle and apoptosis related factors in Huh7/CDDP cells determined by ELISA. * *p* < 0.05, compared to sh-NC + oe-NC; # *p* < 0.05, compared to sh-EZH2 + oe-NC. The cell experiment was repeated three times.

To investigate whether EZH2 promoted HCC progression by miR-381/SETDB1/AKT pathway *in vivo*, we knocked down or overexpressed EZH2 in AKT overexpressed Huh7 cells and analyzed the EHZ2, SETDB1 and AKT pathway-related downstream expression by RT-qPCR and ELISA. We found that sh-EZH2 led to downregulation of EZH2, SETDB1, PT308-AKT, PS473-AKT and PS2448-mTOR accompanied with no changes in the AKT and mTOR expression. In addition, oe-AKT elevated the expression of AKT, mTOR, PT308-AKT, PS473-AKT and PS2448-mTOR without altering that of EZH2 and SETDB1. sh-EZH2 + oe-AKT induced higher expression of AKT, mTOR, PT308-AKT, PS473-AKT and PS2448-mTOR but did not affect that of EZH2 and SETDB1 than sh-EZH2 alone (Figure 7(a,b)).

MTT and Transwell assays indicated that EZH2 silencing significantly repressed HCC cell proliferation and migration, while this suppressive effect was reversed by AKT overexpression or EZH2 overexpression (Figure 7(c,d)). Consistently, we found that EZH2 depletion led to downregulation of KI-67 and N-cadherin and that this downregulation was also rescued by AKT overexpression or EZH2 overexpression (Figure 7(e)). Similarly, we also constructed stable HCC/CDDP cell lines and confirmed the knockdown or overexpression efficiency by RT-qPCR (Figure 7(f)). We next treated these stably transfected cell lines with gradient dose of CDDP and evaluated their sensitivity by MTT assay. The IC_50_ in control cells were 4.703 μg/mL, IC_50_ in EZH2 silenced cells were 1.745 μg/mL and IC_50_ in AKT overexpressed cells were 10.64 μg/mL, IC_50_ in AKT overexpressed + EZH2 silenced cells were 5.087 μg/mL and IC_50_ in EZH2 overexpressed + EZH2 silenced cells were 5.358 μg/mL. Thus, our data revealed that EZH2 silencing significantly increased CDDP sensitivity, while AKT or EZH2 overexpression promoted CDDP resistance (Figure 7(g)).

Furthermore, we observed that EZH2 silencing increased G1 phase-arrested cells and decreased S phase-arrested cells under CDDP treatment, while this effect was also rescued by AKT or EZH2 overexpression (Figure 7(h)). Meanwhile, EZH2 knockdown efficiently downregulated CDK2 expression and promoted cleaved caspase-3 expression in CDDP treated cells and ectopic AKT or EZH2 expression was sufficient to reverse this effect (Figure 7(i)). Collectively, our data revealed that EZH2 promoted the migration, proliferation and chemoresistance of HCC cells by regulating the miR-381/SETDB1/AKT axis.

### 3.8. EZH2 promotes HCC cell tumorigenesis and chemoresistance by regulating the miR-381/SETDB1/AKT axis in vivo

**Figure 8.**
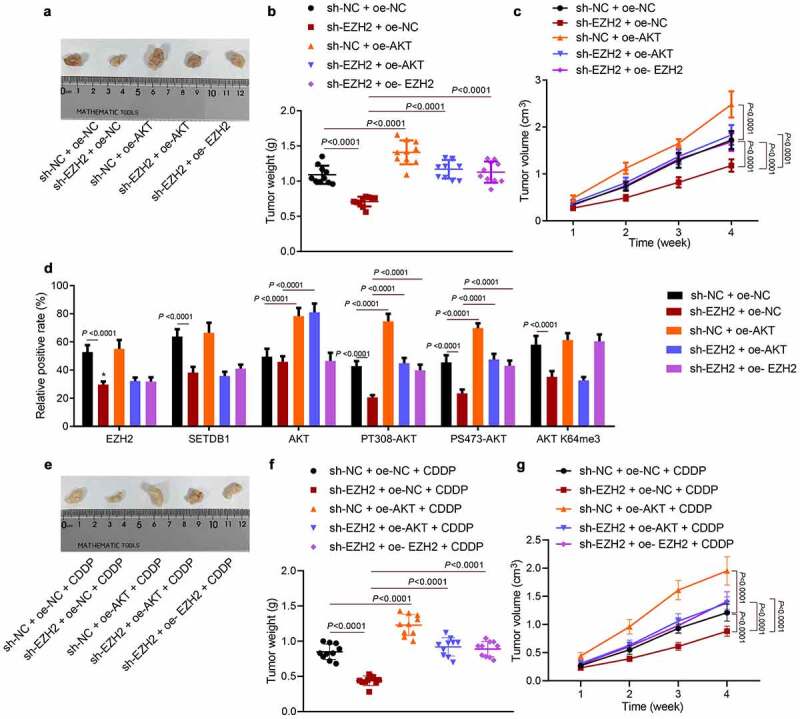
EZH2 promotes HCC cell tumorigenesis and chemoresistance via the miR-381/SETDB1/AKT axis *in vivo*. a, representative photos of xenograft tumors. b, statistic data of tumor weight, * *p* < 0.05, compared to sh-NC + oe-NC; # *p* < 0.05, compared to sh-EZH2 + oe-NC. c, statistical data of tumor volume, * *p* < 0.05, compared to sh-NC + oe-NC; # *p* < 0.05, compared to sh-EZH2 + oe-NC. d, EZH2, SETDB1, AKT, AKT K64me3, PT308-AKT, and PS473-AKT expression in tumor tissues determined by IHC, * *p* < 0.05, compared to sh-NC + oe-NC; # *p* < 0.05, compared to sh-EZH2 + oe-NC. e, representative photos of xenograft tumors under CDDP treatment, * *p* < 0.05, compared to sh-NC + oe-NC + CDDP; # *p* < 0.05, compared to sh-EZH2 + oe-NC + CDDP. f, statistical data of tumor weight. g, statistical data of tumor volume, * *p* < 0.05, compared to sh-NC + oe-NC + CDDP; # *p* < 0.05, compared to sh-EZH2 + oe-NC + CDDP. n = 10 for mice in each group.

Finally, we moved to investigate whether EZH2/miR-381/SETDB1 regulated HCC cell tumorigenesis and CDDP resistance by AKT pathway *in vivo*. EZH2 silencing significantly suppressed tumor weight and volume while this effect was reversed by ectopic AKT expression or by up-regulation of EZH2 (Figure 8(a-c)). Consistent to the *in vitro* data, IHC results showed that sh-EZH2 significantly reduced the expression of EZH2, SETDB1, AKT K140me3, PT308-AKT, PS473-AKT and K64me3 AKT without affecting total AKT levels, while ectopic AKT expression restored the expression of AKT, PT308-AKT, and PS473-AKT without affecting EZH2, AKT K64me3 and SETDB1 expression (Figure 8(d)). Furthermore, we utilized CDDP to treat the xenograft model mice, observed their tumor morphology and recorded tumor size and weight (Figure 8(e-g)). The results revealed that tumor volume and weight were much smaller with sh-EZH2 treatment in comparison with control mice under CDDP treatment, while this effect was abolished by AKT or EZH2 overexpression. In summary, EZH2 could promote HCC cell tumorigenesis and chemoresistance by regulating the miR-381/SETDB1/AKT axis *in vivo*.

### 3.9. miR-381 targeted SETDB1 and inhibited AKT pathway, thus delaying the tumorigenesis in vivo

To further verify the regulatory effect of miR-381 on SETDB1-regulated AKT pathway in promoting tumorigenesis *in vivo*, we constructed a tumor model by establishing miR-381-mimic or oe-NC-treated Huh7 cell lines, recorded the weight and growth of subcutaneous tumors in each group of mice every week. The results revealed that overexpression of miR-381 significantly reduced the tumor weight and volume (Figure S1A-C). RT-qPCR results showed that the expression of miR-381 in xenograft tumors was significantly augmented after treatment of miR-381 mimic (Figure S1D).

Next, IHC and ELISA results showed that the positive rates of SETDB1, AKT K64me3, PT308-AKT and PS473-AKT were decreased after overexpressing miR-381, while the total AKT expression was not significantly different (Figure S1E, F). The above results demonstrated that miR-381 inhibited SETDB1 expression and silenced the AKT pathway, thereby arresting the tumorigenesis *in vivo*.

## 4. Discussion

**Figure 9.**
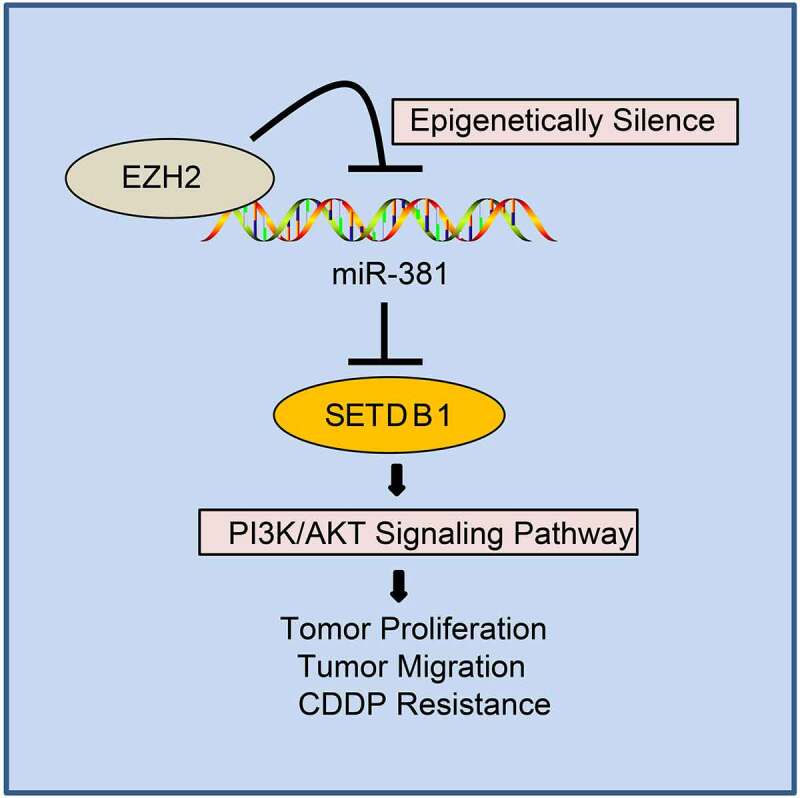
EZH2 regulates AKT pathway via the miR-381/SETDB1 axis to promote occurrence and chemoresistance of HCC.

Accumulating evidence has been highlighted that liver carcinogenesis is driven by the accumulation of various genetic and epigenetic changes [28]. In the current study, we found highly expressed SETDB1 and EZH2 in HCC through microarray-based gene expression profiling. Through a series of *in vitro* and *in vivo* experiments, we provided evidence that EZH2 suppressed miR-381 expression by promoting H3K27me3 activity on its promoter to facilitate SETDB1 expression, thereby activating the AKT pathway to promote hepatocarcinogenesis and chemoresistance (Figure 9).

Our data reveals SETDB1 expression to be aberrantly higher in clinical HCC tissues and *in vitro* cell liens. Notably, we find that depletion of SETDB1 suppresses HCC progression and chemoresistance, accompanied with reduced AKT activity. Furthermore, we demonstrate that SETDB1 promotes HCC cell proliferation, migration, chemoresistance and tumorigenesis *in vivo* by activating AKT via methylating on the K64 site. In line with our study, a previous report also demonstrates higher SETDB1 in HCC cells [29]. Aberrant activation of PI3K-AKT is commonly found in diverse cancer types, including HCC [30]. Constituent activation of AKT has been reported to indicate poor clinical outcome [31]. Moreover, high distant metastatic capacity and chemoresistance are commonly found in tumor cells with constituently activated AKT [8,9]. Notably, a recent report revealed that SETDB1 promoted AKT activation to promote malignant melanomas progression by enhancing its methylation on K64 [10].

A previous report has revealed that miR-381 directly targets and suppresses SETDB1 to inhibit breast cancer cell proliferation [17]. Consistently, our microarray-based gene expression profiling results also identified SETDB1 to be a direct downstream target of miR-381. In contrast to the high expression of SETDB1 in HCC, miR-381 was found to be downregulated in HCC tissues and cell lines. Furthermore, we found that ectopic miR-381 expression significantly suppressed SETDB1 expression and thereby repressed AKT activation. Consistent with our findings, decreased miR-381 was also found in one previously published study [26]. Moreover, partly in line with our study, SETDB1 was confirmed to be a target of miR-381-3p [17]. The question thus emerges how miR-381 is downregulated in HCC cells. EZH2, a methyltransferase and core component of the PRC2 complex, has been reported to be highly expressed in diverse cancer types, including HCC [32–34]. EZH2 is a well-known oncogene and has been commonly recognized as a biomarker for many types of cancers [35,36]. For instance, Xu X et al. reports that EZH2 is recruited to the p21 and E-cadherin promoter to silence their expression by LINC00978 in HCC, which accelerated HCC progression [37]. Meanwhile, EZH2 is also reported to suppress ATOH8 transcription to reduce HCC sensitivity to cisplatin treatment [38]. More importantly, EZH2 has been reported to suppress miR-381 expression to contribute to cisplatin resistance in breast cancer in an epigenetic manner [19]. Our data revealed that EZH2 had aberrantly high expression in HCC specimens and *in vitro* cell lines. Moreover, a dramatically decreased expression of SETDB1 and inactivation of AKT pathway was accompanied by a significant increase of miR-381 expression in EZH2 silenced HCC cells. Meanwhile, EZH2 depleted HCC cells showed less proliferation rates, metastatic capacity, and tumorigenesis potential. Consistent with former studies, EZH2 suppressed miR-381 expression by promoting H3K27me3 modification on its promoter. More importantly, our study uncovers that EZH2 regulates HCC cell proliferation, migration, chemoresistance and tumorigenesis *in vivo* by regulating PI3K-AKT pathway.

## 5. Conclusion

Taken together, our present study demonstrated that highly expressed EZH2 in HCC suppressed miR-381 expression by catalyzing an H3K27me3 modification on its promoter regions. As a consequence, SETDB1, a direct downstream target of miR-381, was upregulated in turn. Due to high expression of SETDB1, AKT K64 tri-methylation levels were significantly higher in HCC tissues, which contributed to its constituent activation. Finally, aberrant activation of the AKT pathway promoted HCC cell proliferation, migration, chemoresistance, and tumorigenesis *in vivo*. Our study not only provides a new biomarker for HCC diagnosis but also supplies a potential drug target to enhance chemotherapy efficiency.

## Supplementary Material

Supplemental Material

## Data Availability

The authors confirm that the data supporting the findings of this study are available within the article [and/or] its supplementary materials.

## Research highlights

1. SETDB1 activates AKT pathway to promote HCC progression and CDDP resistance.
2. miR-381 directly targets SETDB1 and inactivates the AKT pathway in HCC cells.
3. EZH2 silences miR-381 and upregulates the expression of SETDB1 in HCC cells.
4. EZH2 induces tumorigenesis and chemoresistance via miR-381/SETDB1/AKT axis.
5. This study provides new targets for the treatment of HCC.
