# Supplementary material for: Enhancer of zeste homolog 2 promotes hepatocellular cancer progression and chemoresistance by enhancing protein kinase B activation through microRNA-381-mediated SET domain bifurcated 1
Source: Bioengineered. 2022 Feb 19;13(3):5737–55. doi: 10.1080/21655979.2021.2023792 (PMC8974146; doi:10.1080/21655979.2021.2023792)
Supplement: Supplemental Material [file KBIE_A_2023792_SM0919.zip › supplementary/Supplementary Tables.docx]

**Table S1** Characteristics of HCC patients.

| Characteristics | Patients (n = 52) | |
| --- | --- | --- |
| Age (years) | ≤ 50 | 23 |
|  | > 50 | 29 |
| Gender | Male | 39 |
|  | Female | 13 |
| Tumor size (cm) | ≥ 5 | 18 |
|  | < 5 | 34 |
| TNM stage | Ⅰ | 19 |
|  | Ⅱ | 20 |
|  | Ⅲ | 13 |
| Cirrhosis | Yes | 33 |
|  | No | 19 |
| Hepatitis (HBV or HCV) | Negative | 21 |
|  | Positive | 31 |

Notes: HCC, hepatocellular carcinoma; TNM, tumor node metastasis; HBV, hepatitis B virus; HCV, hepatitis C virus.

**Table S2** Primer sequences used for RT-qPCR.

| Genes | Primer Sequence |
| --- | --- |
| SETDB1 (human) | F: AATCTTCCCGGCCTACAGAA |
|  | R: ATAGCTTCACGGAGCTTCTG |
| EZH2 (human) | F: TTGTGGAGTTGGTGAATGCC |
|  | R: ATCTCGGTGATCCTCCAGAT |
| GAPDH (human) | F: TTCGACAGTCAGCCGCATCTT |
|  | R: CCCAATACGACCAAATCCGTT |
| U6 (human) | F: CTCGCTTCGGCAGCACA |
|  | R: GTGCAGGGTCCGAGGT |
| miR-381 (human) | F: TTCTGTAAAGGACTGGGGCC |
|  | R: GTGCAGGGTCCGAGGT |

Notes: SETDB1, SET domain bifurcated 1; EZH2, enhancer of zeste homolog 2; GAPDH, glyceraldehyde-3-phosphate dehydrogenase; miR-381, microRNA-381.
