# Supplementary figures and images for: Enhancer of zeste homolog 2 promotes hepatocellular cancer progression and chemoresistance by enhancing protein kinase B activation through microRNA-381-mediated SET domain bifurcated 1
Source: Bioengineered. 2022 Feb 19;13(3):5737–55. doi: 10.1080/21655979.2021.2023792 (PMC8974146; doi:10.1080/21655979.2021.2023792)

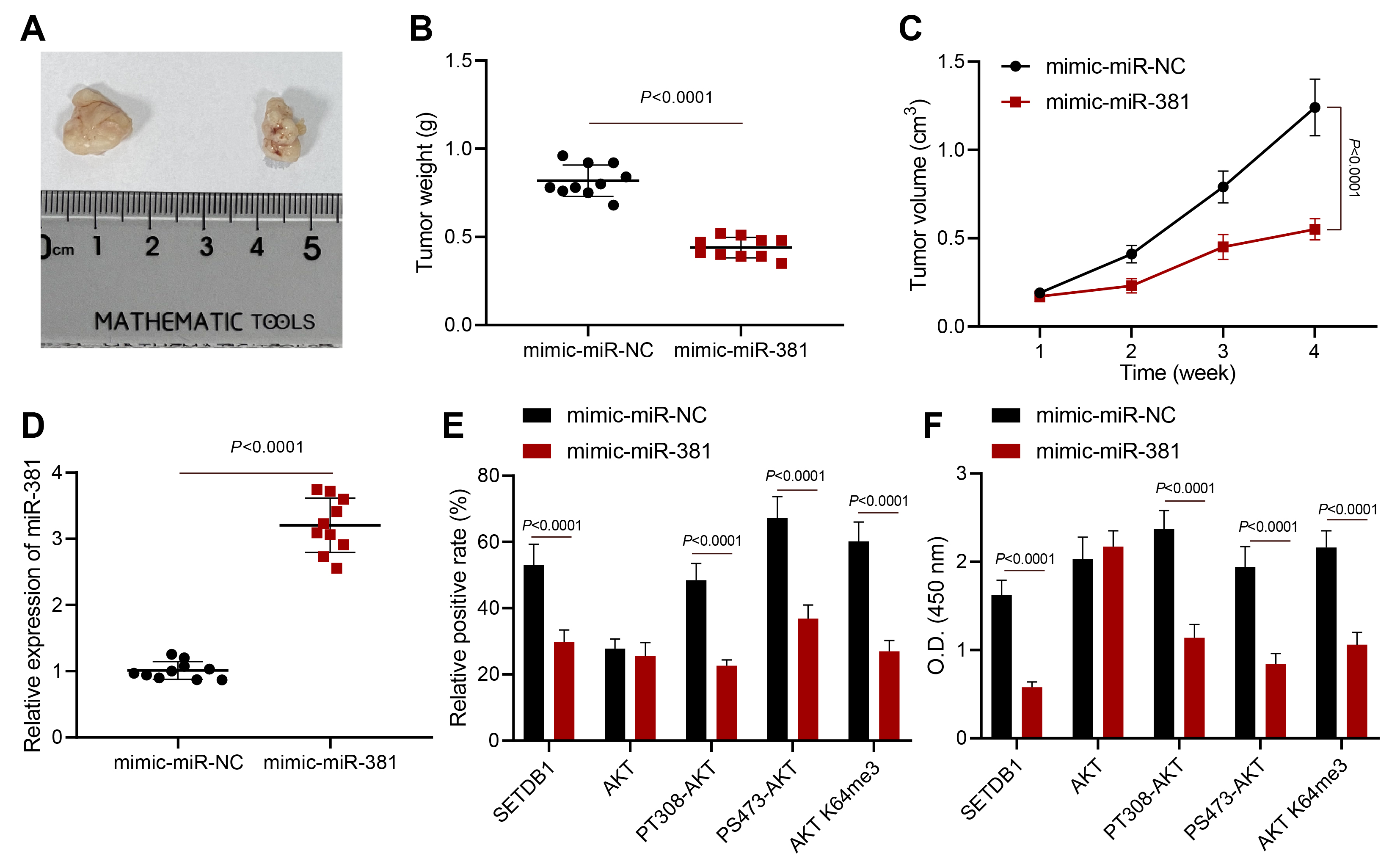

Supplement: Supplemental Material [file KBIE_A_2023792_SM0919.zip › supplementary/Figure S1.jpg]
